# Voltammetric Sensor Based on SeO_2_ Nanoparticles and Surfactants for Indigo Carmine Determination

**DOI:** 10.3390/s22093224

**Published:** 2022-04-22

**Authors:** Liya Kavieva, Guzel Ziyatdinova

**Affiliations:** Analytical Chemistry Department, Kazan Federal University, Kremleyevskaya 18, 420008 Kazan, Russia; liyakavieva@mail.ru

**Keywords:** nanoparticles, surfactants, electrochemical sensors, voltammetry, colorants, indigo carmine, pharmaceutical analysis

## Abstract

Indigo carmine is a widely used colorant in the food and pharmaceutical industry a high concentration of which can lead to a wide range of negative effects on human health. Therefore, colorant contents have to be strictly controlled. SeO_2_-nanoparticle-modified glassy carbon electrodes (GCE) have been developed as a voltammetric sensor for indigo carmine. Various types and concentrations of surfactants have been used as reagents for the stabilization of SeO_2_ nanoparticle dispersions and as electrode surface co-modifiers. An amount of 1.0 mM cationic cetylpyridinium bromide (CPB) provides the best response of the indigo carmine on the modified electrode. The electrodes were characterized by cyclic voltammetry, chronoamperometry, and electrochemical impedance spectroscopy (EIS). SeO_2_ nanoparticle–CPB-modified electrodes show 4.2-fold higher electroactive area vs. GCE as well as a dramatic 5043-fold decrease in the electron transfer resistance indicating effectivity of the modifier developed. The surface-controlled electrooxidation of indigo carmine proceeds irreversibly (α_a_ = 0.46) with the participation of two electrons and two protons. A linear dynamic range of 0.025–1.0 and 1.0–10 µM of indigo carmine were obtained with the detection and quantification limits of 4.3 and 14.3 nM, respectively. The practical applicability of the sensor was successfully shown on the pharmaceutical dosage forms.

## 1. Introduction

The rapid development in nanomaterials opens great opportunities for chemical sensor fabrication, in particular electrochemical ones. Non-metal oxide nanoparticles are of interest among different types of nanomaterials. Nevertheless, the attention is mainly paid to silica nanoparticles (both solid and mesoporous) to which the diverse applications such as sorbents [1,2], semiconductors [3], catalysts [4], and sensitive layers in electrochemical sensors [5,6,7,8,9,10] are reported. Further development in this field is the application of SeO_2_ nanoparticles as an electrode surface modifier due to their excellent stability, high charge transfer, and superior electrochemical specific capacitance [11]. The electrocatalytic effect of SeO_2_ nanoparticles has been shown on the example of a long stable water splitting process [11]. Two electrochemical sensors based on the SeO_2_ nanoparticles have been reported to date. The first one is based on the lotus-flower-like SeO_2_-decorated reduced graphene oxide nanocomposite and used for the amperometric determination of quercetin [12]. It should be noted that electrocatalytic effect is also provided by reduced graphene oxide. Furthermore, the nanocomposite synthesis is a multistage time-consuming process. Another sensor consisted of the glassy carbon electrode modified with SeO_2_ nanoparticles and hexadecyltriphenylphosphonium bromide has been developed for the sensitive voltammetric quantification of carminic acid in candies [13]. Thus, further development of electrochemical sensors based on the SeO_2_ nanoparticles is of practical interest.

Colorants of various classes are widely used in the food, pharmaceutical, and cosmetic industries. Their contents in real samples have to be strictly controlled due to the dose-dependent negative health effects [14]. The majority of colorants can undergo electroreduction or/and electrooxidation allowing their electrochemical determination which is favorable vs. other methods as they are highly sensitive, simple, reliable, cost-effective, and can be miniaturized and realized outside the laboratory.

**Figure 1:**
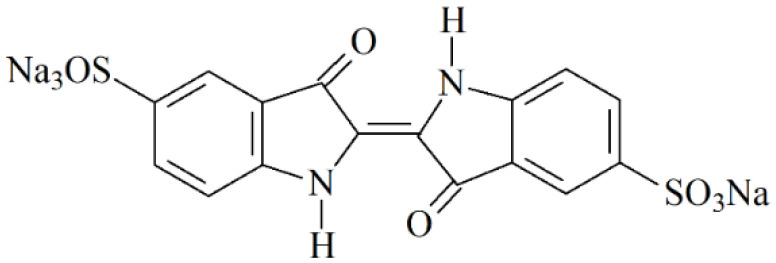
Indigo carmine structure.

The blue colorant indigo carmine (disodium [2(2′)E]-3,3′-dioxo-1,1′,3,3′-tetrahydro[2,2′-biindolylidene]-5,5′-disulfonate, E132) (Figure 1) is of interest among a wide range of colorants. It is used in the food and pharmaceutical industry [15] as well as in medicine for diagnostic purposes [16]. Nevertheless, the overdose of indigo carmine can lead to side effects in the liver, central nervous system, kidneys, and eyes, as well as show mutagenic effects leading to oncogenesis [17]. Therefore, the acceptable daily intake of 5.0 mg kg^−1^ d^−1^ is reported for the indigo carmine [17], and its contents in real samples have to be monitored as part of the quality control procedure.

**Figure 2:**
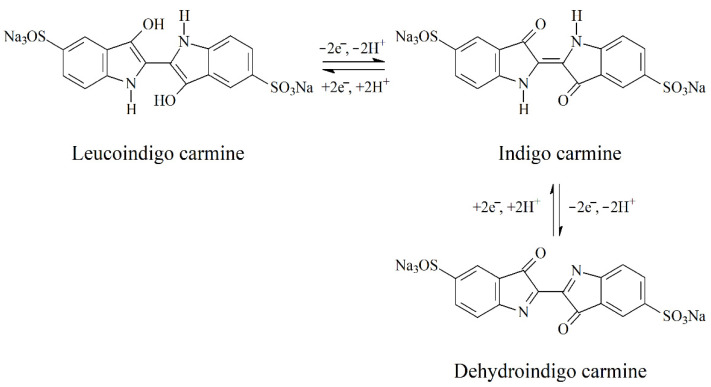
Redox behavior of indigo carmine.

Indigo carmine is an electroactive colorant able to perform the reduction at the electrode surface with the formation of leucoindigo carmine and to the oxidation to dehydroindigo carmine (Figure 2) [18]. Both processes involve two electrons’ and two protons’ transfer.

The colorant reduction has been studied at the silver-based mercury film [19] and screen-printed [20] electrodes. The oxidation of indigo carmine at the platinum [21], screen-printed carbon [22] electrodes, and cathodically pretreated boron-doped diamond electrode [23,24] has been reported. Recently, a wide range of voltammetric sensors based on 4-(4-nitrophenilazo)N-benzyl,N-ethylaniline [25], electropolymerized arginine [26] and glycine [27], zinc porphyrins and zinc phthalocyanine [28], combination of poly(glutamic acid) [29] and chiral amine bis(phenolate) boron complex with multiwalled carbon nanotubes [30] modified electrodes have been developed for the quantification of indigo carmine. Modification of the electrode surface provides significant improvement in the analytical characteristics of the colorant compared to bare electrodes, although limits of detection and linear dynamic ranges obtained for alternating current voltammetric and flow-amperometric detection modes are comparable to the modified electrodes. Other electrochemical sensors with modified surfaces show characteristics comparable to each other that can be further improved. It should be mentioned that there are no data regarding metal and non-metal oxide nanoparticle-based electrochemical sensors for indigo carmine.

The current work is focused on the development of a novel voltammetric sensor for indigo carmine based on the glassy carbon electrode (GCE) modified with SeO_2_ nanoparticles and surfactants. The effect of surfactant nature and concentration on the voltammetric characteristics of indigo carmine was studied. SeO_2_ nanoparticles dispersed in cationic cetylpyridinium bromide provided the best response. The electrooxidation parameters of the indigo carmine were evaluated using cyclic voltammetry. A highly sensitive voltammetric sensor was developed for colorant quantification and successfully tested on pharmaceutical dosage forms.

## 2. Materials and Methods

### 2.1. Reagents

Indigo carmine of analytical grade was obtained from Sigma-Aldrich (Steinheim, Germany). Its standard 10 mM solution was prepared by dissolving an accurately weighed portion in distilled water. An exact dilution was used for the preparation of less concentrated solutions.

SeO_2_ powder of 99.9% purity from Aldrich (Saint Louis, MO, USA) was used. The 1.0 mg mL^−1^ homogeneous dispersions of SeO_2_ nanoparticles in surfactants aqueous solutions of various concentrations were obtained by sonication for 20 min in WiseClean WUC-A03H ultrasonic bath (DAIHAN Scientific Co., Ltd., Wonju-si, Korea).

Cationic cetylpyridinium bromide (CPB) of 98% purity (Aldrich, Steinheim, Germany) and cetyltrimethylammonium bromide (CTAB) (99% from Acros Organics, Geel, Belgium), anionic sodium dodecyl sulfate (SDS) of Ph. Eur. grade (Panreac, Barselona, Spain) and N-lauroylsarcosine sodium salt (LSS) (97% from Aldrich, Steinheim, Germany), and non-ionic Brij^®^ 35 (Acros Organics, Geel, Belgium) and Triton X-100 from Aldrich (Steinheim, Germany) were used as dispersive agents. Their 1.0 mM standard solutions were prepared by the dissolution of the exact weighed portion of the substance in distilled water.

Ascorbic acid (99% from Sigma, Steinheim, Germany), DL-menthol (95% from Sigma-Aldrich, Steinheim, Germany), aspartame (98% from Sigma-Aldrich, Steinheim, Germany), benzydamine hydrochloride (Sigma-Aldrich, Steinheim, Germany), riboflavin (98% from Sigma-Aldrich, Steinheim, Germany), and quinoline yellow (95% from Acros Organics, Geel, Belgium) were used in the interference study. Their 1.0 mM solutions in water were prepared by dissolution of the accurately weighed portion.

Other reagents were of c.p. grade. Distilled water was used for the measurements. The experiments were carried out at laboratory temperature (25 ± 2 °C).

### 2.2. Apparatus

Potentiostat/galvanostat μAutolab Type III (Eco Chemie B.V., Utrecht, The Netherlands) with GPES 4.9.005 software was used for the voltammetric and chronoamperometric measurements. Electrochemical impedance spectroscopy (EIS) was performed on the potentiostat/galvanostat Autolab PGSTAT 302N with FRA 32M module (Eco Chemie B.V., Utrecht, The Netherlands) and NOVA 1.10.1.9 software. A 10 mL glass electrochemical cell consisted of a glassy carbon electrode (GCE) with 7.07 mm^2^ geometric surface area (CH Instruments, Inc., Bee Cave, TX, USA) or SeO_2_ nanoparticles modified GCE as working electrode, a silver-silver chloride 3.5 M KCl reference electrode, and a platinum counter electrode was used.

An “Expert-001” pH meter (Econix-Expert Ltd., Moscow, Russia) equipped with the glass electrode was used for pH measurements.

High-resolution field emission scanning electron microscope Merlin^TM^ (Carl Zeiss, Oberkochen, Germany) operated at the 5 kV accelerating voltage and 300 pA emission current was used for the electrode surface characterization.

### 2.3. Procedures

#### 2.3.1. Sensor Fabrication

The GCE was carefully polished with alumina (0.05 µm) on a polishing cloth, rinsed with acetone, and distilled water. Electrode modification was performed by drop-casting of 4 μL of SeO_2_ nanoparticles dispersion on the surface of GCE and evaporation to dryness for 15 min at room temperature.

#### 2.3.2. Electrochemical Measurements

Voltammetric measurements were performed in the phosphate buffer (PB) of various pH. After fivefold scanning of the potential in the supporting electrolyte, an aliquot of the indigo carmine solution (5.0–50 μL) was added to the electrochemical cell. Cyclic voltammograms were registered within the potential range of 0.1 to 1.0 V at the scan rate of 100 mV s^−1^. Anodic differential pulse voltammograms were recorded from 0.30 to 0.70 V. The pulse parameters were varied. To calculate the oxidation currents in the differential pulse mode, baseline correction using GPES 4.9.005 software (Eco Chemie B.V., Utrecht, The Netherlands) under the moving average algorithm was used.

Chronoamperometry was performed at a potential of 0.45 V in the presence of 1.0 mM K_4_[Fe(CN)_6_] in 0.1 M KCl. The electrolysis time was 75 s.

Electrochemical impedance spectroscopy was performed in the presence of 1.0 mM K_4_[Fe(CN)_6_]/K_3_Fe(CN)_6_ in 0.1 M KCl in the frequency range of 10 kHz–0.04 Hz (9 points per order of magnitude) with an applied sine potential amplitude of 5 mV at a polarization potential of 0.22 V.

Spectrophotometric measurements were performed on the spectrophotometer PE-5300 (NPO Ecros, Saint Petersburg, Russia).

#### 2.3.3. Evaluation of the Electroactive Surface Area of the Electrodes

Cyclic voltammetry and chronoamperometry of 1.0 mM K_4_[Fe(CN)_6_] in 0.1 M KCl were applied for the estimation of electrodes electroactive surface area. Due to the irreversible oxidation of hexacyanoferrate(II) ions on GCE, chronoamperometric data and Cottrell equation [31] (Equation (1)) were used.

(1) I=nFAcD12π−12t−12,

where *I* is the current (A), *n*—the number of transferred electrons, *F*—the Faraday constant (C mol^−1^), *A*—the electrode surface area (cm^2^), *c*—concentration (mol cm^−3^), *D*—diffusion coefficient (cm^2^ s^−1^) and *t*—the electrolysis time (s). For 1.0 mM K_4_[Fe(CN)]_6_ in 0.1 M KCl, *n* = 1 and *D* = 7.6 × 10^−6^ cm^2^ s^−1^ at 298 K.

Electroactive surface area of the SeO_2_ nanoparticles modified GCE was calculated using cyclic voltammetry data and Randles-Sevcik equation [31] (Equation (2)) as far as a reversible oxidation of redox probe was observed.

(2) Ip=π12χpn32F32AcD12(RT)−12υ12,

where *I*_p_ is a peak current (A), $\chi_{p}$—normalized current for sweep experiments with a reversible system, *R*—the universal gas constant (J mol^−1^ K^−1^), *T*—temperature (K) and υ—potential scan rate (V s^−1^). Other variables had the same meaning as in Equation (1).

#### 2.3.4. Pharmaceutical Dosage Forms Analysis

Commercially available pharmaceutical dosage forms containing indigo carmine as colorant (vitamin tablets, lozenges for symptomatic local treatment for the relief of pain and irritation of the mouth and throat, and capsules of non-steroidal anti-inflammatory drug) were used as real samples. The average mass of lozenges, tablets, or capsules shells was preliminarily measured. Then, five lozenges, tablets, or capsules shells of known mass were dissolved in distilled water using a 50 mL flask. In the case of the vitamin tablets, the solution was filtered after the full dissolving of the tablets’ coverage containing the colorant.

PB pH 5.0 was placed into the electrochemical cell and five differential pulse voltammograms were recorded. Then, an aliquot portion of the sample solution (50–150 μL) was added and differential pulse voltammograms were registered from 0.30 to 0.70 V at a modulation amplitude of 100 mV, a modulation time of 25 ms, and a potential scan rate of 10 mV s^−1^.

Spectrophotometric measurements were performed in 2 cm cuvettes at 610 nm vs. distilled water using a standard addition method.

#### 2.3.5. Statistical Treatment of the Data

The measurement results were statistically treated for five replicates at a confidence level of 0.95. All data were expressed as the X ± ΔX with X as average value and ΔX as coverage interval. *F*- and *t*-tests were used for the validation of the developed and independent methods.

The detection and quantification limits were calculated as 3SD_a_/*b* and 10SD_a_/*b*, respectively, where SD_a_ was the standard deviation of the calibration graph intercept and *b*—the calibration graph slope.

Regression analysis was performed using the OriginPro 8.1 software (OriginLab, Northampton, MA, USA).

## 3. Results

### 3.1. Voltammetric Characteristics of Indigo Carmine at the Bare and SeO_2_-Nanoparticle-Modified Electrodes

**Figure 3:**
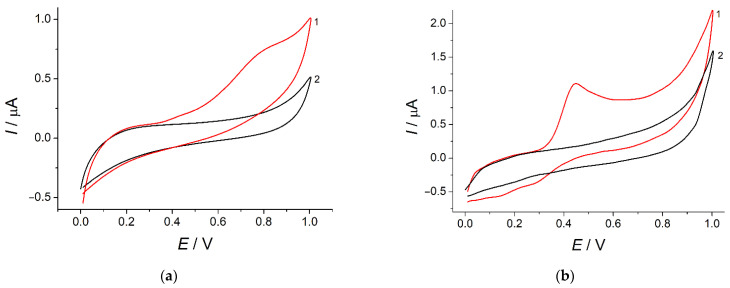
Cyclic voltammograms of 50 µM indigo carmine (curve 1) in PB pH 7.0 (curve 2): (**a**) bare GCE; (**b**) SeO_2_–CPB/GCE. Potential scan rate is 100 mV s^–1^.

Indigo carmine has shown an irreversible weakly pronounced oxidation step at 0.78 V in PB pH 7.0 at the bare GCE (Figure 3, curve 2). The oxidation currents of 76 ± 3 nA for 50 µM concentration indicate a low sensitivity of the response and its inapplicability to the analytical purposes. To solve this problem, electrode surface modification with SeO_2_ nanoparticles was used.

The application of surfactants as dispersive agents and stabilizers for nanoparticles dispersion preparation has been successfully shown for electrochemically inert metal oxide nanoparticles such as CeO_2_ [32,33] and SnO_2_ [34,35]. Furthermore, surfactants act as electrode surface co-modifiers providing organic compounds preconcentration at the electrode surface [36,37,38,39]. The mechanism of surfactant–analyte interactions can vary depending on the nature of both compounds. Thus, dispersions of SeO_2_ particles in surfactant media of various nature and concentrations were used. SeO_2_ nanoparticles are formed in the dispersion and act as electrode surface modifier as scanning electron microscopy confirms (see Section 3.2). Similar behavior has been reported for the SiO_2_ nanoparticles [13,40].

**Figure 4:**
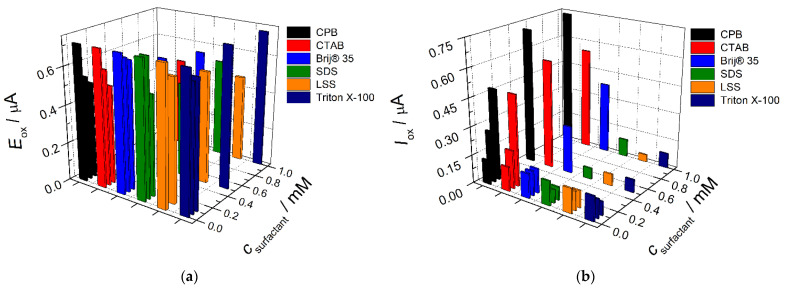
Effect of surfactants on the voltammetric characteristics of 50 µM indigo carmine at the SeO_2_–Surfactant/GCE: (**a**) effect on the oxidation potential; (**b**) effect on the oxidation currents. Supporting electrolyte is 0.1 M phosphate buffer, pH 7.0.

The effect of surfactants’ nature and concentration in the SeO_2_ nanoparticles dispersion on the voltammetric behavior of indigo carmine was studied under conditions of cyclic voltammetry. Cationic (CPB and CTAB), anionic (SDS and LSS), and nonionic surfactants (Brij^®^ 35 and Triton X-100) in the concentration range of 0.050–1.0 mM were used. The changes of indigo carmine voltammetric characteristics are presented in Figure 4. The oxidation potential is significantly decreased as the surfactant concentration increases independently of the surfactant type (Figure 4a). This means the increase in the electron transfer rate for the modified electrodes due to the presence of surfactant. The only exclusion is Triton X-100 for which almost no effect is observed that is probably caused by the presence of polyethylene oxide chain in surfactant structure affecting interaction with the analyte. The oxidation currents of indigo carmine are changed in different ways depending on the surfactants’ nature (Figure 4b).

Anionic SDS and LSS significantly depress the oxidation peak in the whole concentration range that is explained by electrostatic repulsion between negatively charged at pH 7.0 surfactant and indigo carmine. Non-ionic surfactants show a different effect on the indigo carmine oxidation currents. Brij^®^ 35 up to 0.10 mM level does not affect the indigo carmine response while 0.50–1.0 mM concentration of surfactant leads to the increase in the colorant oxidation current. Triton X-100 shows similar to anionic surfactants effect, i.e., significant decrease in the indigo carmine oxidation currents. Cationic surfactants in the whole concentration range provide a statistically significant (1.6–5.7-fold) increase in the oxidation currents of indigo carmine vs. those at the bare GCE. The data obtained allow to conclude that electrostatic interactions play a major role in the carminic acid response. As shown on cationic CPB and CTAB, the hydrophobic interactions also contribute to the improvement in the voltammetric characteristics of indigo carmine. The more pronounced effect in the case of CPB can be explained by the possibility of π-π stacking between the aromatic rings of surfactant and indigo carmine molecules.

The best response of indigo carmine was registered at the electrode modified with SeO_2_ nanoparticles dispersed in 1.0 mM CPB (Figure 1, curve 3) that was used in further investigations. The increase in the oxidation currents is caused by the increase in the effective surface area of the modified electrode (Section 3.2) as well as preconcentration of the colorant at the electrode surface via electrostatic and hydrophobic interactions with a surfactant as confirmed in further investigations (Section 3.3). Furthermore, two worse pronounced reduction steps are appeared in the case of modified electrode that means the improvement in the electrochemical system reversibility.

It should be noted that the synergetic effect of both SeO_2_ nanoparticles and CPB provides an excellent response of the electrode towards indigo carmine. This suggestion is confirmed by the application of CPB-modified electrode providing approximately a 2.0-fold increase in indigo carmine oxidation currents. Furthermore, the reproducibility of the electrode response is insufficient due to the possibility of partial leaching of CPB from the electrode surface after insert in the supporting electrolyte.

### 3.2. Microscopic and Electrochemical Characterization of the Electrodes

**Figure 5:**
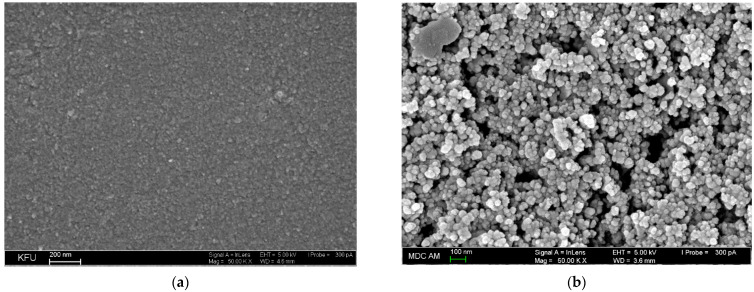
Electrodes surface morphology obtained by scanning electron microscopy: (**a**) bare GCE; (**b**) SeO_2_–CPB/GCE.

Scanning electron microscopy was applied for the characterization of GCE and SeO_2_–CPB/GCE surface (Figure 5). Bare GCE demonstrates a relatively smooth surface of insignificant roughness (Figure 5a). SeO_2_ nanoparticles dispersed in 1.0 mM CPB form porous coverage with the spheroid particles of 39–55 nm that are uniformly distributed at the GCE surface (Figure 5b). Microscopic data confirm the successful immobilization of SeO_2_ nanoparticles at the GCE surface.

**Figure 6:**
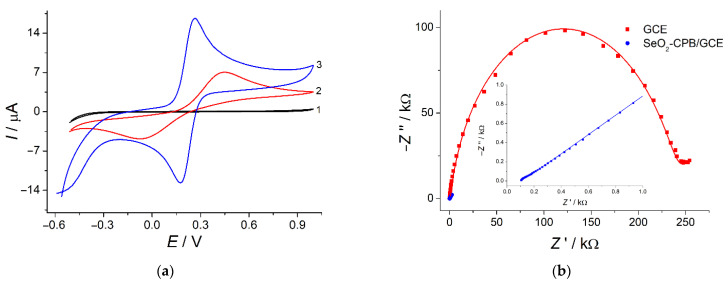
(**a**) Cyclic voltammograms for 1.0 mM [Fe(CN)_6_]^4−^ at the bare GCE (curve 2) and SeO_2_–CPB/GCE (curve 3) in 0.1 M KCl (curve 1), potential scan rate is 100 mV s^−1^; (**b**) Experimental (points) and fitted (lines) Nyquist plots for bare GCE and SeO_2_–CPB/GCE in the presence of 1.0 mM [Fe(CN)_6_]^4−/3−^ in 0.1 M KCl. *E* = 0.22 V; frequency range, 10 kHz—0.04 Hz; amplitude, 5 mV.

The electrode surface modification leads to the increase in the surface area due to the porous structure. The electroactive surface area is also 4.2-fold increased vs. bare GCE (37.4 ± 0.2 vs. 8.9 ± 0.2 mm^2^, respectively) as confirmed by electrochemical data for the electrooxidation of hexacyanoferrate(II) ions in 0.1 M KCl. Furthermore, the application of SeO_2_ nanoparticles dispersion in CPB as electrode surface modifier provides significant improvement in the redox probe system reversibility (Figure 6a, curve 3) vs. bare GCE that indicates the increase in the electron transfer rate on the modified electrode. These data are fully coincided with electrochemical impedance spectroscopy investigation (Figure 6b).

A dramatic decrease in the semicircle diameter in the high frequencies region for the modified electrode indicates significant improvement in the electron transfer vs. GCE. This is also confirmed by electrochemical spectra fitting (Table 1) using Randles equivalent cell consisting of electrolyte resistance (*R*_s_), constant phase element (*Q*), charge transfer resistance (*R*_ct_) and Warburg impedance (*W*) [41]. Charge transfer resistance for the modified electrode is 5043-fold lower than for bare GCE that means increase in the electron transfer rate. The constant phase element value for SeO_2_-nanoparticle-modified electrode is 26.3-fold higher than for bare GCE that can be explained by the increase in the electrode surface total charge due to the presence of CPB. Fitting error (χ^2^ parameter) does not exceed 0.04 confirming accuracy of the equivalent electrical circuit applied. Thus, electrochemical data confirm effectivity of SeO_2_ nanoparticles dispersion in CPB as a novel type of the electrode surface modifiers for the creation of electrochemical sensors.

### 3.3. Indigo Carmine Electrooxidation Parameters

**Figure 7:**
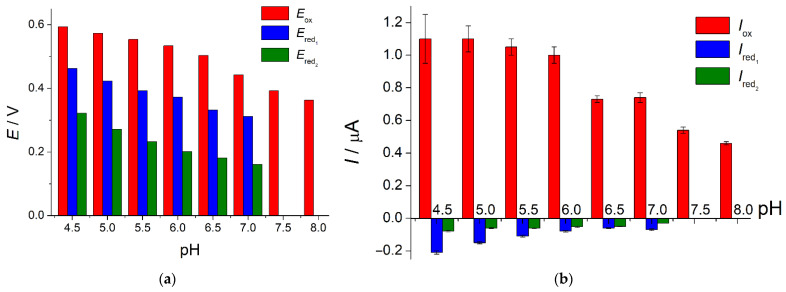
Effect of phosphate buffer pH on the voltametric characteristics of 50 µM indigo carmine at the SeO_2_–CPB/GCE: (**a**) the changes of the oxidation potential; (**b**) the changes of the oxidation currents.

Electrochemical oxidation of indigo carmine at the SeO_2_–CPB/GCE and quantitative characteristics of the process were evaluated using cyclic voltammetry. Firstly, the effect of phosphate buffer pH in the range of 4.5–8.0 on the voltametric characteristics of colorant was studied (Figure 7).

Oxidation of indigo carmine proceeds irreversibly in the whole pH range. There are two weak reduction steps on the cyclic voltammograms at the 4.5–7.0 pH. In spite of the presence of reduction steps on the cathodic branches of voltammograms, indigo carmine oxidation is irreversible as far as the redox peak potential separation is close to 150 mV and currents ratio is significantly less than 1.0. Colorant oxidation/reduction potentials are cathodically shifted with the increase in the phosphate buffer pH. Thus, protons participate in the indigo carmine electrooxidation. The anodic currents at pH 4.5 and 5.0 are the same and then start to decrease as pH is grown. Reduction currents are gradually decreased with the pH increase up to their full disappearance in a basic medium. Phosphate buffer of pH 5.0 has been chosen for further investigations as it provides the highest oxidation currents of indigo carmine and their better reproducibility in comparison to pH 4.5.

**Figure 8:**
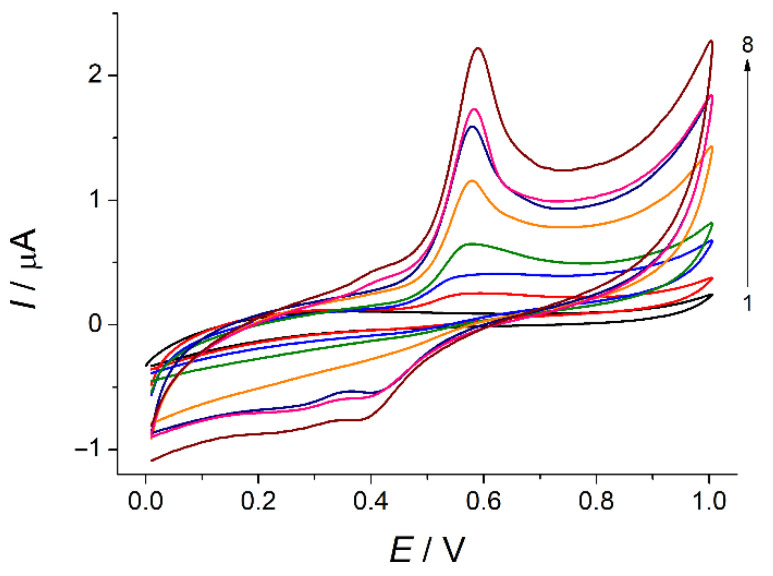
Cyclic voltammograms of 50 µM indigo carmine at the SeO_2_–CPB/GCE at the various potential scan rates: 5 (curve 2), 10 (3), 25 (4), 50 (5), 75 (6), 100 (7), and 150 (8) mV s^−1^ in phosphate buffer pH 5.0 (curve 1).

The effect of potential scan rate in the range of 5–150 mV s^−1^ on the voltammetric response of indigo carmine was evaluated (Figure 8). There is a well-defined oxidation peak of indigo carmine at 0.574 V which is insignificantly increased with the potential scan rate growth.

The peak currents are linearly increased with the potential scan rate in the range of 5–75 mV s^−1^ (Equation (3), Figure S1a) and the slope of the plot ln*I* = f(lnυ) is close to 1.0 (Equation (4), Figure S1b) that confirms the surface-controlled electrooxidation. Similar behavior has been reported for polyarginine- [26] and polyglycine-modified [27] carbon paste electrodes. Further increase in the scan rate leads to the deviation from linearity that is also typical for the surface-controlled processes. Thus, the data obtained confirms preconcentration of indigo carmine at the electrode surface due to the interaction with CPB.

*I*_p_ [µA] = (0.025 ± 0.002) + (0.0134 ± 0.0005) υ [mV s^−1^]     *R*^2^ = 0.99995, (3)

ln*I*_p_ [µA] = (2.38 ± 0.07) + (0.91 ± 0.02) lnυ [V s^−1^]     *R*^2^ = 0.99855, (4)

The electrooxidation parameters of indigo carmine have been found. The α_a_*n* of 1.01 has been calculated from Equation (5) [31].

∆*E*_1/2_ = 62.5/α_a_*n*, (5)

The anodic transfer coefficient α_a_ = 0.46 has been calculated using Equation (6) [31].

(6) $$I_{p}=\frac{\alpha_{a}n_{\alpha }FQ\upsilon }{2.718RT},$$

Thus, the number of electrons participating in the indigo carmine electrooxidations equals to two. Based on the data obtained, the two electron and two proton irreversible electrochemical process with formation of dehydroindigo carmine is suggested that agree well to he reported for other modified electrodes [25,26,27].

Equation (7) has been applied for the calculation of the indigo carmine surface coverage (*Γ*) [31].

(7) $$I_{p}=\frac{n\alpha_{a}n_{\alpha }F^{2}A\upsilon \Gamma }{2.718RT},$$

Indigo carmine surface coverage is (1.8 ± 0.1) × 10^−10^ mol cm^−2^.

### 3.4. Indigo Carmine Sensing Using SeO_2_ Nanoparticles Modified Electrode

**Figure 9:**
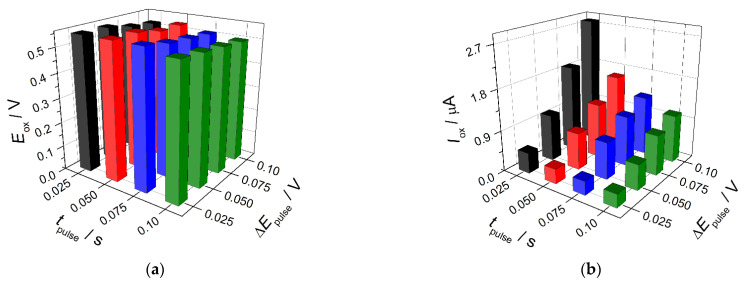
Effect of pulse parameters on the voltammetric characteristics of 50 µM indigo carmine at the SeO_2_–CPB/GCE in phosphate buffer pH 5.0: (**a**) the changes of the oxidation potential; (**b**) the changes of the oxidation currents.

The electrode developed was applied as a voltammetric sensor for indigo carmine. A differential pulse voltammetry was chosen for the improvement in the colorant response sensitivity. The changes of the pulse parameters have shown that the best parameters of the indigo carmine analytical signal are achieved using pulse width of 0.10 V and pulse height of 0.025 s (Figure 9).

**Figure 10:**
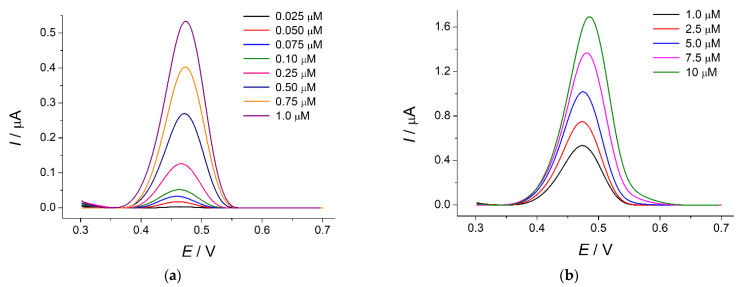
Baseline-corrected differential pulse voltammograms of indigo carmine at the SeO_2_–CPB/GCE in phosphate buffer pH 5.0: (**a**) 0.025–1.0 µM; (**b**) 1.0–10 µM. Pulse width is 0.10 V, pulse height is 0.025 s, and potential scan rate is 10 mV s^−1^.

Under these conditions, the sensor gives well-resolved response to indigo carmine at 0.47 V (Figure 10).

The oxidation currents are linearly dependent on the colorant concentration in the ranges of 0.025–1.0 (Equation (8), Figure S2a) and 1.0–10 µM (Equation (9), Figure S2b) with the detection and quantification limits of 4.3 and 14.3 nM, respectively.

*I* [µA] = (−0.0089 ± 0.008) + (55.9 ± 0.2) × 10^4^
*c* [M]     *R*^2^ = 0.99994, (8)

*I* [µA] = (0.417 ± 0.006) + (127.9 ± 0.9) × 10^3^
*c* [M]     *R*^2^ = 0.99979, (9)

The calibration graph slopes indicate the high sensitivity of the sensor developed. The obtained analytical characteristics of indigo carmine are among the best ones reported to date (Table 2).

The most impressive characteristics for the trace analysis of indigo carmine were obtained at the graphite and diamond paste dot microsensors modified with zinc porphyrins and phthalocyanine [28]. Nevertheless, the modifier can show an electrocatalytic effect to a wide range of compounds contained in the real samples. The selectivity test covers only alizarin red and alizarin blue while other synthetic colorants with the related structure and typical constituents are out of consideration. The practical applicability of the sensors was shown on the spiked wastewater only.

Another advantage of the sensor developed is a simple one-step fabrication as well as easy and express preparation of the modifier.

The sensor response is of high accuracy (99–100%) and reproducibility (RSD values of 0.65–2.1%) that is shown on the indigo carmine model solutions (Table 3). The indigo carmine electrooxidation product is adsorbed at the sensor surface leading to significant decrease (30–50%) in the colorant oxidation currents. Therefore, sensor surface renewal is required after each measurement. On the other hand, the single usable screen-printed electrodes based on the modifier developed can be fabricated.

The sensor response robustness was evaluated by changing reference and working electrodes, orthophosphoric acid and sodium hydroxide used for the phosphate buffer preparation, dispersion of SeO_2_ nanoparticles in CPB as sensor surface modifier. The RSD values of the oxidation currents of 0.50 µM indigo carmine does not exceed 2.5% confirming high robustness of the developed sensor response.

The selectivity of indigo carmine response was studied at the 0.50 µM level. The most typical interferences such as inorganic ions (1000-fold excess of K^+^, Mg^2+^, Ca^2+^, NO_3_^−^, Cl^−^ and SO_4_^2−^ ions) and saccharides (100-fold excess of glucose, sucrose, rhamnose) are electrochemically inactive in the potential range under consideration and do not affect indigo carmine response. Ascorbic acid is oxidized at 0.16 V and does not show interference effect up to 10 µM. Pharmaceutical dosage forms containing indigo carmine as a colorant contain other active and auxiliary principles that can affect the sensor response. Menthol, aspartame, benzydamine hydrochloride, and riboflavin do not oxidize under conditions of the experiment and do not show interference effect. Indigo carmine is often used together with quinoline yellow; therefore, it was tested as a potential interference. Quinoline yellow does not show the oxidation step in the potential range under consideration. Thus, the SeO_2_-nanoparticle-based sensor shows an excellent selectivity towards indigo carmine.

### 3.5. Application of the Sensor in Pharmaceutical Analysis

**Figure 11:**
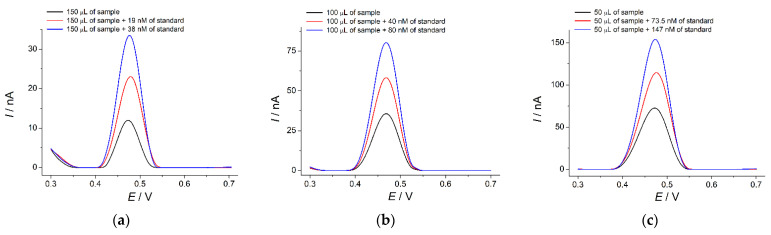
Baseline-corrected differential pulse voltammograms of pharmaceutical dosage forms in the absence and in the presence of indigo carmine additions at the SeO_2_–CPB/GCE in phosphate buffer pH 5.0: (**a**) vitamin tablets; (**b**) lozenges for symptomatic local treatment of the mouth and throat; (**c**) capsules shell of non-steroidal anti-inflammatory drug. Pulse width is 0.10 V, pulse height is 0.025 s, and potential scan rate is 10 mV s^−1^.

The sensor developed was applied for the quantification of indigo carmine in various pharmaceutical dosage forms (vitamin tablets, lozenges for symptomatic local treatment of the mouth and throat, and capsules of non-steroidal anti-inflammatory drug). All samples show a well-defined oxidation peak at 0.47 V corresponding to indigo carmine that is also confirmed by the standard addition method (Figure 11). The recovery values of 99.3–100% (Table 4) indicate the absence of the matrix effects in the pharmaceutical dosage forms analysis.

Indigo carmine quantification results using a SeO_2_-nanoparticle-based sensor and an independent spectrophotometric method are presented in Table 5. The results of both methods agree with each other as well as with the labelled amount. *t*- and *F*-tests are less than critical, which confirms the absence of systematic errors in the determination and uniform precision of both methods.

## 4. Conclusions

A novel highly sensitive voltammetric sensor was developed for indigo carmine quantification. SeO_2_ nanoparticles dispersed in cationic surfactant CPB was used as a sensitive layer for the first time. A dual role of CPB as dispersive agent for SeO_2_ nanoparticles and sensor surface co-modifier providing preconcentration of indigo carmine was shown. Such an approach to the sensor surface modification provided high electroactive area and improvement in the electron transfer rate, as well as sufficient conductivity of the sensor.

The analytical characteristics of indigo carmine on the sensor developed are among the best ones reported to date for other electrochemical approaches. Furthermore, the sensor is simple in fabrication and gives a selective, reproducible, robust, and reliable response to indigo carmine. The possibility of the direct analysis of indigo carmine in different types of pharmaceutical dosage forms using the sensor developed confirms its versatility. The results obtained significantly expand the analytical capabilities of the electrochemical sensors in pharmaceutical quality control.

## Figures and Tables

**Table 1:** Electrochemical impedance parameters of the electrodes (*n* = 5; *p* = 0.95).

Electrode	*R*_s_ (Ω)	*R*_ct_ (kΩ)	*Q* (µΩ^−1^)	*n*	*W* (µΩ^−1^)	χ^2^
GCE	93 ± 3	232 ± 7	0.198 ± 0.003	0.898	62 ± 3	0.040
SeO_2_–CPB/GCE	104 ± 5	0.046 ± 0.005	5.2 ± 0.3	0.936	394 ± 6	0.032

**Table 2:** Figures of merit for the electrochemical sensors for indigo carmine.

Sensor	Detection Mode	Limit of Detection (µM)	Linear Dynamic Range (µM)	Ref.
Silver-based mercury film electrode	AdSV ^1^	-	0–0.21	[19]
Screen-printed electrode	Flow amperometry	0.033	0.05–100	[20]
Screen-printed carbon electrodes	CV ^2^	0.19	0.5–100	[22]
	DPV ^3^	0.17	0.2–20	
	SWV ^4^	0.34	0.7–10	
	ACV ^5^	0.032	0.06–0.7	
Cathodically pretreated boron-doped diamond electrode	SWV	0.058	0.5–84.1	[23]
	FIA-MPA ^6^	0.040	0.07–1.0	[24]
4-(4-Nitrophenilazo)N-benzyl,N-ethylaniline—CPE ^7^	DPV	0.36	1–100	[25]
Polyarginine/CPE	DPV	0.0253	0.2–1.0 and 1.5–3.5	[26]
Polyglycine/CPE	CV	0.011	2–10 and 15–60	[27]
Zinc tetraphenylporphyrin—Graphite PDM ^8^	DPV	3.87 × 10^−8^	1.00 × 10^−6^–1.00 × 10^−4^	[28]
Zinc tetranaphthoporphyrin—Graphite PDM		2.30 × 10^−6^	1.00 × 10^−4^–1.00 × 10^−2^	
Zinc tetrasulphophenylporphyrin—Graphite PDM		1.65 × 10^−6^	1.00 × 10^−6^–1.00 × 10^−4^	
Zinc phthalocyanine—Graphite PDM		4.26 × 10^−5^	1.00 × 10^−3^–1.00 × 10^−1^	
Zinc tetraphenylporphyrin—Diamond PDM		0.0796	0.010–10	
Zinc tetranaphthoporphyrin—Diamond PDM		1.20 × 10^−5^	1.00 × 10^−4^–1.00 × 10^−2^	
Zinc tetrasulphophenylporphyrin—PDM		0.0012	0.010–10	
Zinc phthalocyanine—Diamond PDM		0.0333	0.010–1.0	
Poly(glutamic acid)/MWNT ^9^—based paste electrode	DPV	0.36	5.0–50	[29]
Chiral amine bis(phenolate) boron complex containing N,N-diethyl-*p*-phenylenediamine—carboxylated MWNT/GCE	SWV	0.019	0.1–30	[30]
SeO_2_ nanoparticles—CPB/GCE	DPV	0.0043	0.025–1.0 1.0–10	Current

^1^ Adsorptive stripping voltammetry. ^2^ Cyclic voltammetry. ^3^ Differential pulse voltammetry. ^4^ Square-wave voltammetry. ^5^ Alternating current voltammetry. ^6^ Flow injection analysis with multiple pulse amperometric detection. ^7^ Carbon paste electrode. ^8^ Paste dot microsensor. ^9^ Multi-walled carbon nanotubes.

**Table 3:** Indigo carmine determination in model solutions (*n* = 5; *p* = 0.95).

Added (µg)	Oxidation Current (µA)	Found (µg)	RSD (%)	Recovery (%)
0.0583	0.005 ± 0.0002	0.058 ± 0.001	1.0	99 ± 1
0.233	0.046 ± 0.002	0.23 ± 0.01	1.8	100 ± 2
1.75	0.41 ± 0.01	1.74 ± 0.03	1.3	99 ± 2
11.7	1.06 ± 0.03	11.7 ± 0.3	2.1	100 ± 2
23.3	1.7 ± 0.03	23.3 ± 0.2	0.65	99.8 ± 0.8

**Table 4:** Indigo carmine recovery in pharmaceutical dosage forms (*n* = 5; *p* = 0.95).

Sample	Spiked (nM)	Found (nM)	RSD (%)	Recovery (%)
Vitamin tablets	0	38 ± 2	3.9	
	19	57 ± 2	2.4	100
	38	76 ± 1	1.5	100
Lozenges	0	80 ± 2	1.9	
	40	120 ± 3	2.4	100
	80	159 ± 3	0.72	99.4
Capsules	0	147 ± 7	3.8	
	73.5	219 ± 8	1.4	99.3
	147	292 ± 8	1.8	99.3

**Table 5:** Quantification of indigo carmine in pharmaceutical dosage forms (*n* = 5; *p* = 0.95).

Sample Type	№	Labelled Amount (mg)	Found by Voltammetry (mg)	RSD (%)	Found by Spectrophotometry (mg)	RSD (%)	*t*-Test ^1^	*F*-Test ^2^
Vitamin tablets		-	0.0047 ± 0.0003	4.4	0.0051 ± 0.0003	4.5	1.97	1.21
Lozenges	1	0.015	0.0150 ± 0.0004	2.3	0.0148 ± 0.0003	1.5	0.976	2.07
	2	0.031	0.0311 ± 0.0009	2.3	0.0313 ± 0.0009	2.4	0.559	1.12
Capsules		-	0.055 ± 0.002	3.7	0.053 ± 0.002	2.7	1.31	0.810

^1^*t*_crit_ = 2.31 at α = 0.05 and *f* = 8. ^2^ *F*_crit_ = 6.39 at α = 0.05 and *f*_1_ = *f*_2_ = 4.

## Data Availability

The data presented in this study are available in the electronic Supplementary Materials.

## Supplementary Materials

The following are available online at https://www.mdpi.com/article/10.3390/s22093224/s1, Figure S1. Effect of potential scan rate on the oxidation currents of 50 µM indigo carmine at the SeO_2_–CPB/GCE in phosphate buffer pH 5.0: (a) Plot of oxidation current vs. potential scan rate; (b) Plot of the Napierian logarithm of oxidation current vs. Napierian logarithm of the potential scan rate, Figure S2. Calibration plots of indigo carmine at the SeO_2_–CPB/GCE in phosphate buffer pH 5.0: (a) in the concentration range of 0.025–1.0 µM; (b) in the concentration range of 1.0–10 µM.
