# Supplementary material for: Voltammetric Sensor Based on SeO2 Nanoparticles and Surfactants for Indigo Carmine Determination
Source: Sensors (Basel). 2022 Apr 22;22(9):3224. doi: 10.3390/s22093224 (PMC9103650; doi:10.3390/s22093224)
Supplement: Supplementary file 1 [file sensors-22-03224-s001.zip › sensors-1658244-supplementary.pdf]

Electronic supplementary data

Voltammetric Sensor Based on SeO_2 Nanoparticles and Surfactants for Indigo Carmine Determination

Liya Kavieva and Guzel Ziyatdinova

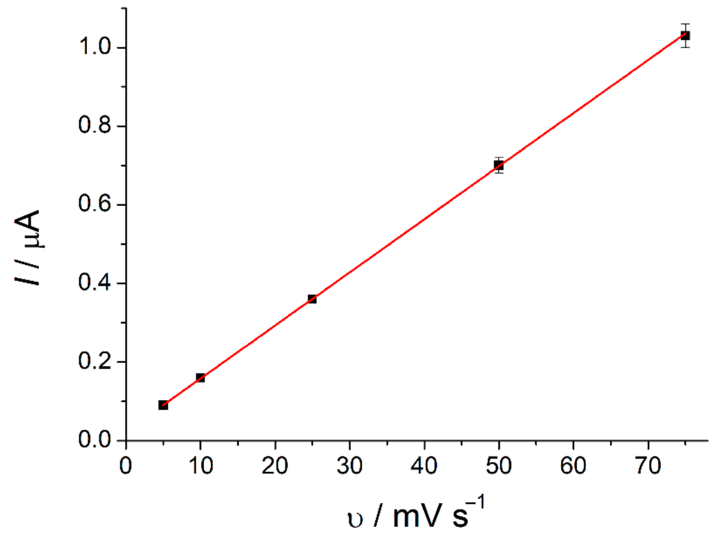

(**a**) (**b**)

Figure S1. Effect of potential scan rate on the oxidation currents of 50 μM indigo carmine at the SeO_2–CPB/GCE in phosphate buffer pH 5.0: (**a**) Plot of oxidation current vs. potential scan rate; (**b**) Plot of the Napierian logarithm of oxidation current vs. Napierian logarithm of the potential scan rate.

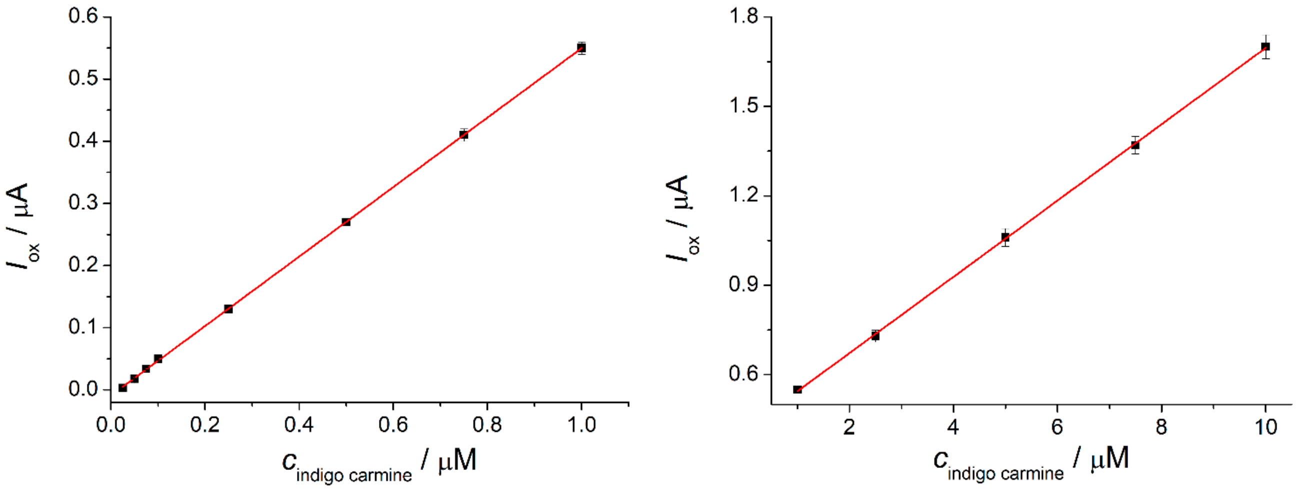

(**a**) (**b**)

Figure S2. Calibration plots of indigo carmine at the the SeO_2–CPB/GCE in phosphate buffer pH 5.0: (**a**) in the concentration range of 0.025–1.0 μM; (**b**) in the concentration range of 1.0–10 μM.
